# Quality of Life and Sexual Satisfaction in the Early Period of Motherhood—A Cross-Sectional Preliminary Study

**DOI:** 10.3390/jcm12247597

**Published:** 2023-12-09

**Authors:** Maria Florkiewicz-Danel, Kornelia Zaręba, Michał Ciebiera, Grzegorz Jakiel

**Affiliations:** 1 Department of Nursing, Faculty of Rehabilitation, Józef Piłsudski University of Physical Education in Warsaw, 00-968 Warszawa, Poland; mariaflorkiewicz@wp.pl; 2 Department of Obstetrics and Gynecology, College of Medicine and Health Sciences (CMHS), United Arab Emirates University (UAEU), Al Ain 17666, United Arab Emirates; 3 2nd Department of Obstetrics and Gynecology, Center of Postgraduate Medical Education in Warsaw, 01-809 Warsaw, Poland; michal.ciebiera@gmail.com; 4 Warsaw Institute of Women’s Health, 00-189 Warsaw, Poland; 5 1st Department of Obstetrics and Gynecology, Center of Postgraduate Medical Education in Warsaw, 01-004 Warsaw, Poland; grzegorz.jakiel1@o2.pl

**Keywords:** fatigue during puerperium, sexuality of women, sexual after childbirth, puerperium, sexuality, quality of life, quality of life after childbirth

## Abstract

The aim of the study was to assess the impact of breastfeeding-related fatigue and family support on the sexuality and quality of life of mothers during early motherhood. A cross-sectional preliminary study was conducted between 1 October 2021 and 15 May 2022 in 65 women being in early postpartum period. We used the authors’ questionnaire developed for the purposes of the study; the Sexual Satisfaction Scale for Women—SSS-W; the Mell–Krat scale for women; and the General Health Questionnaire—GHQ28. A significant negative correlation was found between the age of the patients and the reduction in somatic symptoms (GHQ28 questionnaire) (r = −0.315, *p* = 0.011). Women working professionally achieved significantly higher results in the SSS-W contentment category (r = 0.313, *p* = 0.014). Frequent sexual activity reduced disorders in social functioning (the GHQ28 questionnaire) (r = −0.107, *p* = 0.283). Women who breastfed up to 5 times a day (*p* = 0.033) reached significantly higher SSS-W scores in terms of communication. The partner’s help significantly contributed to higher sexual satisfaction in the aspect of compatibility (*p* = 0.004) and the overall level of satisfaction determined with the SSS-W questionnaire (*p* = 0.016). The presented study suggests that older mothers who are employed and supported by a partner have a higher level of contentment, sexual satisfaction and quality of life.

## 1. Introduction

Sexuality is a complex mechanism that consists of numerous factors such as mental, physical and social factors [1]. However, the sense of sexual satisfaction changes at different periods of a woman’s life—especially in pregnancy [2]. Problems with sexuality may occur at any stage of pregnancy, peaking at 80% in the third trimester, puerperium and early motherhood [3,4]. The situation results from both biological changes occurring in the woman’s body during pregnancy and childbirth, as well as psychological and social changes related to the change of social roles in the relationship and adaptability of the couple [5].

Sexuality and intimacy after childbirth undergo numerous changes, redefining the concept of the partner norm [6]. When facing the challenge of parenthood, the couple focuses on overcoming difficulties associated with caring for a child, which makes sexual activity less important [7]. Therefore, the majority of couples reported a decrease in sexual activity during puerperium compared to the situation before the birth of the first child [8]. Such a trend was found to persist for the first year after delivery [9]. 

Fatigue is a multifaceted phenomenon that concerns the mental, physical and social spheres [10]. It is most commonly defined as “subjective reluctance to continue a task” [11]. Postpartum fatigue is defined as a feeling of being exhausted and overwhelmed associated with a decrease in mental and physical abilities [12]. 

Social support consists of the belief that the people with whom we live and stay will help us in difficult situations that we are experiencing at the moment [13,14]. Family and friends are the main source of emotional, instrumental and informational support for women during early motherhood [15]. The partner and family may improve self-esteem and reduce the level of perceived stress [16]. Notably, lack of support or crisis situations in the relationship may negatively influence the quality of life and sexuality of women [17,18]. 

In connection with numerous sexual disorders reported by patients in the postpartum period and the lack of clear results, we decided to verify the impact of factors related to the postpartum period on sexual disorders and the quality of life after childbirth. The preliminary hypothesis assumed that fatigue associated with the need to get up frequently at night and breastfeeding constituted the main component of sexual disorders among women in early motherhood.

## 2. Materials and Methods

The present results constitute research conducted to define the impact of stress and fatigue on women’s sexuality during early motherhood. In this manuscript, we focused on the psychological aspect of sexuality and the quality of life. 

The study was conducted in accordance with the Declaration of Helsinki and approved by the Bioethics Committee of the Center of Postgraduate Medical Education, Warsaw (Poland) (approval number 72/PB/2018).

This cross-sectional study was conducted in randomly selected patients (simple random sampling) giving birth at a freestanding birth center in the Żelazna Medical Center Ltd. On the second day after childbirth, patients staying in the Obstetric Department were asked for consent to participate in the study. Subsequently, within 6 to 8 weeks after childbirth, they were asked to complete an anonymous questionnaire.

The criteria for inclusion in the study group were as follows:

- Age: 18–45 years;
- Spontaneous pregnancy (without the use of assisted reproductive technology);
- No risk factors in terms of premature birth or a significant pathology of pregnancy.

The criteria for exclusion in the study group were as follows:

- Significant medical complications during childbirth and puerperium (hemorrhages, a significant inflammation, body temperature above 38 °C);
- Hospitalizations during puerperium;
- Use of hormonal contraception;
- Severe chronic diseases or relevant medical history that might affect sexuality (i.e., neoplastic diseases, endocrine diseases, or psychiatric diseases),

A total of 65 women who were recruited between 1 October 2021 and 15 May 2022 were examined. The survey return rate was 79.3%. 

### 2.1. Methodology

The present authors used four tools to collect data from the respondents. The present authors’ questionnaire was developed for the purposes of the study. It mainly concentrated on sociodemographic and medical history and issues concerning sexual activity. Another three surveys were standardized and accredited by the Polish Psychological Society and Polish Sexological Society:

- the Sexual Satisfaction Scale for Women—SSS-W;
- the Mell–Krat scale for women;
- the General Health Questionnaire—GHQ28.

#### 2.1.1. The Present Authors’ Questionnaire

The questionnaire developed for the study consisted of the following five sections: general information, medical history, gynecological and sexual history, obstetric history, and religion. Regarding the demographic data, the respondents were asked about their age, place of residence, education, religion, marital status and information concerning the financial situation of the family. The assessment of the fatigue of the surveyed patients comprised the following factors, which were considered indirect indicators of fatigue: the number of children the respondents had, the frequency of getting up to attend to the child at night, breastfeeding frequency, professional work, support from the partner, family and society.

#### 2.1.2. The Sexual Satisfaction Scale for Women (SSS-W)

The SSS-W scale was developed in the United States by Meston and Trapnell. It is used to assess sexual satisfaction and sexual distress in women [19]. The Polish adaptation of the scale was made by K. Janowski, W. Karłowicz and M. Kubiak. The scale is composed of 18 items and consists of three subscales: contentment, communication and compatibility. The responses are presented on a 5-point Likert scale. In each subscale, the score ranged from 6 to 30. The total score for the entire survey ranged from 18 to 90. The reliability coefficient (Cronbach’s α) of the scale was 0.93 for the overall score [20]. 

#### 2.1.3. The Mell–Krat Questionnaire

The Mell–Krat questionnaire was authored by Mell and Kratochvil to assess the sexual needs and reactions of the subject [21]. The Kromierzynska’s version of the questionnaire by Mell and Kratochvil, consisting of 20 questions, is the adaptation of this tool. The scale measures a number of psychophysiological features related to sexual reactivity, such as the frequency of orgasm, libido, and arousal before intercourse [22]. In the Mell–Krat questionnaire, 1 to 4 points could be scored for each question (the maximum score is 80 points). Women who scored below 55 points were classified as having low sexual reactivity. The reliability coefficient (Cronbach’s α) for the scale is 0.69 [23].

#### 2.1.4. The General Health Questionnaire—GHQ28

The General Health Questionnaire—GHQ28 was developed by Goldberg in 1978 and is used for the overall assessment of the mental health of the respondents. The questionnaire is composed of 4 thematic sections, i.e., somatic symptoms (questions 1–7), anxiety/insomnia (questions 8–14), social dysfunctions (questions 15–21) and severe depression (questions 22–28). Each answer in the GHQ28 questionnaire may score from 0 to 3 points. The maximum score is 84 points. The score of 23/24 is the threshold for the presence of distress. The reliability coefficient (Cronbach’s α) is 0.9–0.95 [24].

### 2.2. Statistics

The data were subjected to statistical analysis using IBM SPSS Statistics 28 (New York, NY, USA, 2021). Statistical significance threshold was assumed at *p* < 0.05. The Shapiro–Wilk test was used to assess whether the data were normally distributed. Intergroup differences regarding quantitative data were assessed using non-parametric tests (due to the lack of the normal distribution of the data)—the Mann–Whitney U test was used in case of two groups, and the Kruskal–Wallis test was used in the case of more groups (Bonferroni test was selected as a post hoc test). The linear relationship between quantitative variables was assessed using the Spearman’s correlation coefficient (due to the lack of normal distribution in the data) and Kendall’s tau-b (for ordinal variables). The responses were converted into the ordinal scale (1 = several times a year, 7 = several times a day) in order to determine the impact of the frequency of intercourse on quality of life and sexual satisfaction. Since the ordinal scale was used, the Kendall tau-b correlation coefficient was selected for statistical purposes.

## 3. Results

### 3.1. Sociodemographic Data

A total of 65 women participated in the study (Table 1). Women aged 25–35 (80%) and urban residents (83.1%) constituted the largest group of the respondents. Most partners of the surveyed women were over the age of 30 (80%). The majority of women (over 80%) as well as their partners (76.9%) had a university degree. Parents of over 52% of the respondents had secondary education. Over 77% of the respondents were married. The majority of women in the study group worked professionally during pregnancy (80%), and over 98% declared a stable financial situation.

#### 3.1.1. Age

A significant negative correlation was shown between the age of the patients and the somatic symptoms, according to the GHQ28 questionnaire (r = −0.315, *p* = 0.011). No significant relationship was demonstrated between the age of the patients and the results scored in the GHQ28 questionnaire in terms of anxiety/insomnia (r = −0.074, *p* = 0.556), social functioning disorders (r = 0.062, *p* = 0.626) or depression (r = −0.164, *p* = 0.191) (Table 2).

The Mell–Krat questionnaire results revealed no impact of age on the sexuality of the respondents (r = −0.042, *p* = 0.755). Similarly, we observed no influence of age on statistically significant changes in the following items of the SSS-W scale: contentment (r = −0.015, *p* = 0.909), communication (r = −0.024, *p* = 0.851), compatibility (r = −0.209, *p* = 0.097) and the total score (r = −0.085, *p* = 0.503) in relation to women’s sexuality.

#### 3.1.2. Professional Work

The Mann–Whitney U test showed no significant differences in the results of the GHQ28 questionnaire in the respondents who worked professionally and who did not work professionally either in terms of somatic symptoms (*p* = 0.276), anxiety and insomnia (*p* = 0.392), social functioning disorders (*p* = 0.465) or depression (*p* = 0.729). 

The Mell–Krat questionnaire results revealed no impact of professional work on the sexuality of the surveyed women (r = 0.113, *p* = 0.396). Regarding the SSS-W scale, no influence was found in terms of communication (r = 0.123, *p* = 0.333), compatibility (r = 0.220, *p* = 0.081) or the total score (r = 0.226, *p* = 0.073). However, women who worked professionally achieved significantly higher sexual satisfaction in the aspect of contentment (r = 0.313, *p* = 0.014) compared to those who did not work professionally (Table 3).

#### 3.1.3. Number of Children

The Kruskal–Wallis test showed no significant differences in the results of the GHQ28 questionnaire in women who had one child, two children or three children or more—either in terms of somatic symptoms (*p* = 0.571), anxiety and insomnia (*p* = 0.384), social functioning disorders (*p* = 0.849) or depression (*p* = 0.169).

The number of children was found to have no impact on sexual satisfaction in the Mell–Krat questionnaire (*p* = 0.099) or in the SSS-W scale in the aspects of contentment (*p* = 0.705), communication (*p* = 0.800), compatibility (*p* = 0.885) and for the general score (*p* = 0.636). 

### 3.2. Indirect Indicators of Fatigue

#### 3.2.1. Frequency of Waking up to Attend to the Child

The Kruskal–Wallis test showed no significant differences in the results of the GHQ28 questionnaire in individuals who woke up at night to attend to the child once or twice, three times or four to six times, either in terms of somatic symptoms (*p* = 0.247), anxiety and insomnia (*p* = 0.592), social functioning disorders (*p* = 0.405) or depression (*p* = 0.061).

Regarding the Mell–Krat questionnaire and the aspects of contentment, communication, compatibility and the total score obtained in the SSS-W scale (*p* > 0.05 in each case), no association was found between the frequency of waking up to attend to the child and sexual satisfaction.

#### 3.2.2. Breastfeeding

The Mann–Whitney U test did not show the impact of breastfeeding on the results of the GHQ28 questionnaire either in terms of somatic symptoms (*p* = 0.516), anxiety and insomnia (*p* = 0.278), social functioning disorders (*p* = 0.957) or depression (*p* = 0.606). Similarly, the Kruskal–Wallis test showed no significant differences regarding the results of the GHQ28 questionnaire depending on the frequency of breastfeeding per day (Table 4).

No effect of breastfeeding on sexual satisfaction was shown in the Mell–Krat questionnaire (*p* = 0.645) or in the SSS-W scale in the aspect of contentment (*p* = 0.882), communication (*p* = 0.062), compatibility (*p* = 0.464) or the total score (*p* = 0.695). However, women who breastfed up to 5 times a day obtained significantly higher SSS-W scores in the communication aspect (*p* = 0.033) compared to women who breastfed 6 to 10 times a day (post hoc test, *p* = 0.036), and 11 times or more (post hoc test, *p* = 0.010).

### 3.3. Partner and Social Support

The Mann–Whitney U test showed no significant differences in the results of the GHQ28 questionnaire in terms of partner assistance with the child in the aspect of somatic symptoms (*p* = 0.464), anxiety and insomnia (*p* = 0.986), social functioning disorders (*p* = 0.203) and depression (*p* = 0.329). 

The Mell–Krat questionnaire (*p* = 0.338) and the SSS-W scale showed no impact of the partner’s assistance in caring for the child on contentment (*p* = 0.058) or communication (*p* = 0.073). However, women whose partner helped with childcare achieved significantly higher sexual satisfaction in the aspect of compatibility (*p* = 0.004) (SSS-W scale) and the overall level of satisfaction determined with the SSS-W questionnaire (*p* = 0.016) (Table 5).

The Mann–Whitney U test showed no significant differences in the results of the GHQ28 questionnaire in women who could count on help with the child from other family members and others apart from the partner: somatic symptoms (*p* = 0.660), anxiety and insomnia (*p* = 0.582), social functioning disorders (*p* = 0.705) and depression (*p* = 0.898). 

Similarly, the Mell–Krat questionnaire (*p* = 0.297) and the SSS-W scale showed no significant differences in women who were helped to care for their child by someone other than their partner (contentment (*p* = 0.645), communication (*p* = 0.341), compatibility (*p* = 0.461) and the total score (*p* = 0.945).

### 3.4. Sexuality and Quality of Life 

Frequent sexual intercourse was associated with a decrease in the results of the GHQ28 questionnaire in the aspect of social functioning disorders (r = −0.107, *p* = 0.283). No relationship was shown between the frequency of intercourse and the results of the GHQ28 questionnaire as regards somatic symptoms (r = −0.107, *p* = 0.283), anxiety and insomnia (r = −0.082, *p* = 0.411) or depression (r = −0.094, *p* = 0.379).

The Mann–Whitney U test showed no effect of the frequency of intercourse on the level of sexual satisfaction determined with the Mell–Krat questionnaire (*p* = 0.871). The Kruskal–Wallis test did not reveal the influence of the frequency of intercourse on the level of sexual satisfaction determined with the SSS-W questionnaire in the aspect of contentment (*p* = 0.525), communication (*p* = 0.321), compatibility (*p* = 0.494) and the total score (*p* = 0.475) in women who reported intercourse at different frequencies. However, an increase in the group of respondents by those who reported intercourse once a month or less might result in obtaining significant differences.

The Mann–Whitney U test showed no significant differences in the results of the GHQ28 questionnaire in individuals who claimed to have oral sex and those who did not in terms of somatic symptoms (*p* = 0.838), anxiety and insomnia (*p* = 0.714), social functioning disorders (*p* = 0.712) or depression (*p* = 0.411). 

No impact of oral sex on sexual satisfaction was shown in the Mell–Krat questionnaire (*p* = 0.080) and the SSS-W scale with regards to compatibility (*p* = 0.709), contentment (*p* = 0.933) and the total score (*p* = 0.261). However, women who had oral sex achieved greater sexual satisfaction as regards communication (SSS-W scale) (*p* = 0.043) (Table 6).

The Mann–Whitney U test showed no significant differences in the results obtained from individuals having anal sex in the GHQ28 questionnaire in the aspect of somatic symptoms (*p* = 0.190), anxiety and insomnia (*p* = 0.055), social functioning disorders (*p* = 0.784) or depression (*p* = 0.887).

Regarding the Mell–Krat questionnaire (*p* = 0.179) and the SSS-W scale, there was no impact of anal sex on sexual satisfaction regarding contentment (*p* = 0.729), communication (*p* = 0.062), compatibility (*p* = 0.645) or the total SSS-W score (*p* = 0.244).

## 4. Discussion

The definition of sexual health assumes a positive and respectful approach to sexuality and sexual relationships and the possibility of pleasurable and safe sexual experiences, as well as freedom from coercion, discrimination and violence [25]. Sexuality and intimacy after childbirth undergo numerous changes, thereby redefining the concept of the partner norm [7,26]. The present study is a broad analysis of the functioning of women in the sexual and psychological sphere during the postpartum period, based on rarely used but valuable research tools. It also highlights factors influencing the quality of the life of women and areas of their functioning requiring special attention. The present study did not demonstrate that breastfeeding and the need to get up frequently at night significantly affected women’s sexuality and quality of life. It only showed significantly higher SSS-W results in terms of communication in women who breastfed up to 5 times a day. A significant negative correlation was found between the age of the patients and the reduction in somatic symptoms (GHQ28 questionnaire). Moreover, women working professionally achieved significantly higher results in the SSS-W contentment category, and frequent intercourse reduced social functioning disorders (the GHQ28 questionnaire). The study did not show that social support had an impact on improving the quality of the life of women during early motherhood. However, it showed an impact of partner assistance on higher sexual satisfaction in the aspect of compatibility and the overall level of satisfaction determined with the SSS-W questionnaire. The study demonstrated that the partner and, particularly, the couple’s compatibility in the sexual sphere were the main factors affecting the quality of a woman’s sexual life. 

Many women expect motherhood to be a joyful time in their lives [27]. At the same time, women giving birth for the first time often feel unprepared for the role of mother. This results in the lack of self-confidence, stress, deterioration of the quality of life and avoidance of sexual contact [28,29]. The number of children was not found to have an impact on sexual satisfaction and the quality of life. Conversely, a study conducted by Mortazava in the third trimester of pregnancy and 8 weeks after childbirth in a group of 357 Iranian women showed that multiparity had a negative impact on the quality of life in young mothers [30]. Therefore, it may be concluded that, compared to the cited study, despite having children and the time devoted to them, Polish women might be fulfilled in the role of mothers, which exerts a positive effect on their sexual activity. 

Sexual health is an important aspect of life at any age in women, and age may undoubtedly affect the quality of life and sexuality [31,32]. A study by Mousavi et al. showed that younger age had a positive influence on the quality of life of women in early motherhood [33]. A study conducted by Boroumandfar et al. in a group of 384 women in the period of 6 to 12 weeks after childbirth showed no effect of age on the sexuality of women after childbirth [34]. Similarly, studies by Zhang, Anzaku, Leigh and Mortazov did not confirm such an association [30,35,36,37,38,39,40,41,42,43,44,45,46,47,48,49,50,51,52,53,54,55,56,57]. Similar results were obtained in the present study, but the largest group (80%) included women between the ages of 25 and 35. Presumably, a study conducted in a larger group of patients aged 35 years and older might reveal significant intergroup differences. Therefore, based on the present research, we may conclude that young people are characterized by more vitality, understood as enhanced physiological capacity and mental state, which is why they can achieve a higher quality of life and sexual satisfaction [38]. 

Numerous women work professionally in early motherhood [39]. Women thus act as a caregiver at home and as a professional in the workplace, which may lead to numerous internal and external conflicts [40]. According to Zhang et al., professional work performed within 6 months after childbirth had a negative impact on women’s sexuality [35]. A study by Karl et al. was conducted on 587 women who were surveyed eight weeks after giving birth. It was shown that the difficulty associated with the need to combine work and private life had a negative influence on their mental health [39]. The problem was particularly linked to insecure working conditions associated with inadequate remuneration in relation to the work performed [40]. The present study revealed no impact of professional work on the quality of life. However, working women reported a higher sense of sexual contentment. This might be due to a multilevel sense of fulfillment—both in the maternal and professional aspect, or the financial independence that professional work gives. Therefore, women may experience a greater sense of self-esteem, which may translate into their sexual satisfaction. However, such an observation might be also the result of a small sample size. Moreover, the results may be hindered by the fact that the majority of women included in the study were well-educated and reported an above-average financial status.

Breastfeeding and childcare are important and socially promoted tasks that pose a challenge to the mother of a newborn child [41]. A study conducted by Anzaku et al. in 340 women up to 14 weeks after childbirth showed no effect of breastfeeding on sexual functioning [36]. Similar results were obtained by Boroumandfar et al. and Saotome et al. [34,42]. A study conducted by Mokhtaryan-Gilani et al. demonstrated that breastfeeding women obtained higher scores in questionnaires assessing the quality of life [43]. The frequency of breastfeeding is an important modifying factor. According to Mousavi et al., frequent breastfeeding had a positive effect on the quality of life of the respondents [33]. Based on the conducted research, it may be concluded that women who breastfeed more often satisfy the need for intimacy, mainly through physical contact with the child, which is why they are less likely to seek intimacy from their partner. Therefore, the frequency of sexual contact might be decreased [41]. However, no relationship between breastfeeding and changes in the sexual sphere was observed in the present study. Seemingly, a study conducted in a larger group of patients might show significant differences between groups.

During early puerperium, the partner is the most important source of both emotional and instrumental support, and satisfaction with the relationship is one of the most important components of the mental health of women after childbirth [44]. Scientific research also indicated the importance of effective communication in the relationship as a protective factor against sexual disorders, especially during puerperium [44]. A study conducted by Emmanuel et al. in 473 women revealed that support from a partner positively influenced a woman’s mental and physical health [45]. Similarly, research conducted by Muise et al. showed higher sexual satisfaction in women receiving partner support and understanding of the need to delay sexual activity [46]. A study by Hipp et al. showed no impact of social support on women’s sexuality after childbirth [1]. A study conducted by McLeish et al. in 47 mothers who received peer support during pregnancy and early parenthood showed that individual peer support could have a positive effect on the mother’s well-being [47]. The present study did not confirm this statement. However, it should be noted that almost all study group women were supported by a partner or family in caring for their child, which probably influenced the results. A study on a larger group of women could show such an association due to the greater variability of responses occurring in larger groups. However, based on the present study, it may be concluded that the support of a partner and family had a positive effect on the quality of life of women during puerperium. Attention should also be paid to the importance of family care assistance, which is characteristic of the Polish culture. It worth adding that institutional support in Poland in the first year is considerable. An employee is granted at least 20 weeks of 100% paid maternity leave. Moreover, fathers are entitled to two weeks of paid paternity leave. The length of maternity leave depends on the number of children born, and may reach up to 37 weeks with the birth of the fifth child or above. Once maternity leave ends, mothers are entitled to 32 weeks (or 34 weeks in case of a multiple birth) of parental leave. This leave may be split between parents [48].

The estimated time of the resumption of sexual intercourse after childbirth varies, de-pending on the methodology and location of the study, ranging from a few days to six months after childbirth [49,50]. Moreover, a decrease in the frequency of intercourse shown in the perinatal period was probably an avoidance mechanism associated with the risk of becoming pregnant again [51,52]. This phenomenon is explained as a protective factor for a newborn child, who is completely dependent on parents [53]. According to Hipp et al., women were more likely to engage in other forms of sexual activity, such as oral or anal sex, for fear that vaginal sex would be painful, as confirmed by studies [1,49]. 

The most commonly reported complaints occurring immediately after delivery include pain and fatigue, as well as vaginal dryness (44%) and sexual desire disorders (44%) occurring in connection with physiological changes developing after childbirth [54,55]. Women also reported reduced self-esteem and concerns about changes in the body early in the puerperium, such as episiotomy-related discomfort, swelling and pain in the perineal region [28,56,57,58]. Vaginal dryness and dyspareunia were found to reduce the quality of life and affect the frequency of sexual contacts [58,59]. This was confirmed by a study by Thompson et al. conducted on the 4th day and 8, 16 and 24 weeks after delivery. The authors reported that perineal pain and sexual problems were much more common in primiparas. Those problems significantly affected perceived sexual satisfaction [60]. Parents also repeatedly reported a decrease in relationship satisfaction and problems in the sexual sphere, such as disorders of desire, arousal and sexual satisfaction [61,62]. The loss of libido is one of the most common sexual dysfunctions in the post-delivery period. Low sex drive was also found to affect the level of sexual satisfaction [63]. Orgasm is individually variable during the postpartum period: it occurs without significant changes; it is less intense; or does not occur. Painful intercourse and lack of orgasm lead to distress and fear of not being able to return to normal sexual activity [64]. A study conducted by Martínez-Galiano et al. in women at 6 weeks after childbirth showed that women who had problems with intercourse associated with discomfort due to bladder and bowel incontinence, dyspareunia and hemorrhoids were less likely to engage in sexual activity and were characterized by a reduced quality of life [65]. Conversely, a study by Saotome conducted in 127 couples showed that sexual dysfunctions assessed from 1 month to 12 months after childbirth did not affect the level of sexual satisfaction.

The present study showed that more frequent intercourse was associated with fewer problems concerning social functioning disorders in relation to the quality of life in women. Moreover, the frequency of intercourse did not affect sexuality in the general sense of the test, although an increase in the number of people in the group who had intercourse less frequently than once a month might show statistical significance. Furthermore, women who had oral sex achieved higher scores in the communication category of the SSS-W questionnaire. Therefore, it may be concluded that women who have sex more often achieve a higher quality of life, and women who have oral sex achieve a higher level of communication with their partner, which is associated with a higher level of intimacy in relationships. In conclusion, frequent intercourse during early motherhood may reduce the stress associated with a new life situation. Therefore, it might contribute to improving the quality of life [66]. Moreover, sexual activity and a strong bond between partners are interrelated, i.e., sexual activity strengthens the bond which, in turn, has a positive effect on sexuality [67].

Over 87% of pregnant women reported fatigue with an increasing tendency equaling 95% during puerperium [68,69]. Fatigue associated with motherhood is observed even up to one year after childbirth [10]. It constitutes an important factor in decreasing libido, reducing the frequency of sexual intercourse, increased risk of postpartum depression and reducing the perceived quality of life [70,71,72,73,74,75]. Battaiah et al. demonstrated that its severity might be influenced by such factors as sleep quality, the age of the respondents, breastfeeding and childcare [76]. Woolhouse indicated the need for night-time childcare as the main cause of the phenomenon [77]. Poor sleep quality was found to increase the incidence of depression, anxiety and cognitive impairment, including memory, attention, cognitive abilities and the ability to regulate emotions [78,79,80]. Moreover, Al Rehail also demonstrated that poor sleep quality decreased the quality of the life of women after childbirth [81]. The birth of a child significantly affects the quality of the parents’ sleep, which has a negative influence on physical and mental well-being [82]. According to available research, the quality of sleep may be influenced by the quality of the relationship with the partner, as well as the marital status of the respondents [83]. Attention should also be paid to the actual number of hours slept and the quality of sleep interrupted by waking up, which may further aggravate mood and quality of life disorders [84]. MacKenzie conducted a study in 203 mothers assessing the correlations of infant sleep quality, parents’ sleep, frequency of intercourse and sexual desire up to 12 months after delivery. It was found that poorer infant sleep was associated with poorer partner sleep quality, translating into a lower frequency of intercourse and lower sexual desire [85]. Rowland also showed that insufficient sleep weakened women’s libido during the postpartum period [73]. A study by Valla et al. revealed that the mothers of children who did not wake up at night achieved a higher quality of life compared to women who woke up at night [86]. The study did not show an effect of the frequency of getting up at night to attend to the child on sexual satisfaction and the quality of life. This was probably related to the fact that almost all women were breastfeeding, which reduced the time of night awakenings resulting from the need to prepare infant formula and feed the baby. Perhaps, the intimate relationship with the child and the feeling of fulfillment as a mother were also likely to improve the quality of life and the quality of sex life. The results confirm the validity of Rosemary Basson’s theory stating that sexual pleasure and satisfaction are not reliant on orgasm, and desire for increased emotional closeness and intimacy or overtures from a partner may predispose a woman to participate in sexual activity [87].

The lack of knowledge and preparation regarding changes in social roles and changes occurring in the sexual sphere may contribute to conflicts in the relationship and the deterioration of the overall quality of the life of women and their partners [88].

### 4.1. Strenghts

The conducted study allowed us to broaden the perspective and analye the functioning of women in the sexual and psychological sphere during the postpartum period. It draws attention to the occasional protective impact of frequent breastfeeding on women’s sexuality and quality of life during early motherhood. It also indicates the significant impact of a partner’s support in caring for the child on the quality of sexual life after childbirth. It shows no impact of organic fatigue resulting from motherhood on women’s sexuality in case of maintaining protective factors such as partner and family support.

### 4.2. Limitations

The small size of the group resulted from the unfavorable time of conducting the study, i.e., the end of the COVID-19 pandemic. Moreover, the birth rate in Poland decreased by 11% at that time. The study was designed as a longitudinal one, and the presented results are only preliminary ones. Difficulties in the recruitment of the group resulted from the fact that the examined patients also had their blood drawn in order to determine the level of hormones.

## 5. Conclusions

The presented study suggests that breastfeeding does not affect the quality of life or the sexual satisfaction of women during early motherhood in women who breastfeed up to 5 times a day. Older mothers with employment who are supported by a partner are characterized by a higher level of contentment, sexual satisfaction and quality of life. It seems that adequate support from the partner and proper communication in the relationship may constitute protective factors for sexual disorders after childbirth. Women maintaining sexual activity are more likely to be characterized by better social functioning and, thus, a better quality of life.

## Figures and Tables

**Table 1:** Sociodemographic data of the respondents (*n* = 65).

Variable	*n*	%
**Level of education**		
Master’s degree	53	81.50%
Bachelor’s degree	3	4.60%
Secondary	9	13.80%
**Level of education—partner**		
Master’s degree	47	72.30%
Bachelor’s degree	3	4.60%
Secondary	15	23.10%
**Level of education—parents**		
Master’s degree	24	36.90%
Bachelor’s degree	6	9.20%
Secondary	34	52.30%
Primary	7	10.80%
**Age**		
Under 25	1	1.50%
25–30	26	40.00%
30–35	26	40.00%
35–40	11	16.90%
40+	1	1.50%
**Age—partner**		
Under 25	1	1.50%
25–30	12	18.50%
30–35	28	41.50%
35–40	13	20%
40+	12	18.50%
**Place of residence**		
Village	8	12.30%
Province capital	45	69.20%
District capital	5	7.70%
Other towns	4	6.20%
No data	3	4.60%
**Marital status**		
Single (never married) woman	14	21.50%
Married	50	76.90%
Divorced	1	1.50%
**Professional work**		
Does not work professionally	12	18.50%
Works professionally	52	80.00%
No data	1	1.50%
**Financial status**		
A significant amount of money	2	3.10%
Enough money	27	41.50%
Average level	34	52.30%
Insufficient amount of money	1	1.50%
No data	1	1.50%
**Frequency of intercourse**		
once a month or less frequently	4	6.10%
several times a month or once a week	25	38.50%
twice a week or more	36	55.40%
**Type of sexual activity (Multiple answers possible)**		
Oral sex	44	67.70%
Anal sex	13	20.00%
Vaginal sex	65	100%
**Number of children**		
1	32	49.20%
2	25	38.50%
3 or more	8	12.30%

**Table 2:** Age and GHQ28 results (*n* = 65).

		Age of the Patient
**GHQ28 somatic symptoms**	r	−0.315
	*p*	0.011
	*n*	65
**GHQ28 anxiety/insomnia**	r	−0.074
	*p*	0.556
	*n*	65
**GHQ28 social functioning disorders**	r	0.062
	*p*	0.626
	*n*	65
**GHQ28 depression**	r	−0.164
	*p*	0.191
	*n*	65

**Table 3:** Professional work and the results of the SSS-W questionnaire, Mell–Krat.

Correlations		
		Professional Work
**SSS-W contentment**	r	0.313 *
	*p*	0.014
	*n*	61
**SSS-W communication**	r	0.123
	*p*	0.333
	*n*	64
**SSS-W compatibility**	r	0.220
	*p*	0.081
	*n*	64
**SSS-W total**	r	0.226
	*p*	0.073
	*n*	64
**Mell–Krat total**	r	0.113
	*p*	0.396
	*n*	59

*: statistical significance.

**Table 4:** The frequency of breastfeeding per day and the results of the SSS-W, Mell–Krat, and GHQ28 questionnaires.

	How Many Times a Day Do You Breastfeed?	*n*	Mean	Median	SD	Min	Max	*p*-Value
**GHQ28 Somatic symptoms**	Up to 5 times	10	7.80	7.00	2.251	6	12	0.802
	6 to 10 times	35	7.37	7.00	3.687	2	18	
	11 times or more	19	7.63	8.00	3.077	3	13	
**GHQ28 Anxiety/insomnia**	Up to 5 times	10	10.70	10.00	6.075	2	19	0.087
	6 to 10 times	35	6.91	7.00	3.399	1	15	
	11 times or more	19	6.00	6.00	3.127	2	12	
**GHQ28 Social functioning disorders**	Up to 5 times	10	9.80	9.50	4.211	3	17	0.421
	6 to 10 times	35	8.46	8.00	4.252	1	28	
	11 times or more	19	9.11	8.00	4.446	2	19	
**GHQ28 Depression**	Up to 5 times	10	2.40	1.00	3.204	0	8	0.490
	6 to 10 times	35	1.29	0.00	1.994	0	7	
	11 times or more	19	0.84	0.00	1.167	0	4	
**Mell–Krat total**	Up to 5 times	10	52.20	55.00	8.929	32	61	0.355
	6 to 10 times	33	53.21	54.00	7.968	29	70	
	11 times or more	19	49.74	49.00	10.386	31	72	
**SSS-W Contentment**	Up to 5 times	10	21.20	21.50	3.259	16	26	0.134
	6 to 10 times	35	21.54	23.00	4.598	10	27	
	11 times or more	19	18.79	20.00	5.308	10	26	
**SSS-W Communication**	Up to 5 times	10	26.40	29.00	4.300	18	30	0.033
	6 to 10 times	35	22.63	23.00	4.479	10	30	
	11 times or more	19	20.95	22.00	5.662	12	30	
**SSS-W Compatibility**	Up to 5 times	10	22.30	23.50	6.219	12	30	0.226
	6 to 10 times	35	25.17	26.00	4.422	14	30	
	11 times or more	19	23.37	24.00	4.833	12	30	
**SSS-W total**	Up to 5 times	10	69.90	72.00	11.445	49	86	0.229
	6 to 10 times	35	69.34	72.00	11.368	44	86	
	11 times or more	19	63.11	66.00	13.395	40	82	

**Table 5:** Partner assistance vs. SSS-W questionnaire results, (*n* = 65).

	Partner’s Help with the Child	*n*	Mean	Median	SD	Min	Max	The Mann–Whitney U Test	
								Statistics	*p*-Value
**SSS-W contentment**	Yes	60	21.15	22.50	4.433	10	27	−1.894	0.058
	No	5	15.80	14.00	6.017	10	26		
**SSS-W communication**	Yes	60	23.10	23.00	4.856	10	30	−1.791	0.073
	No	5	18.80	16.00	6.221	13	29		
**SSS-W compatibility**	Yes	60	24.78	25.50	4.491	12	30	−2.841	0.004
	No	5	17.40	19.00	4.393	12	23		
**SSS-W total**	Yes	60	69.03	71.50	11.041	41	86	−2.402	0.016
	No	5	52.00	49.00	15.017	40	78		
**Mell–Krat total**	Yes	60	52.33	52.50	8.827	29	72	39.284	0.338
	No	5	48.80	47.00	9.418	38	62		

**Table 6:** Oral sex vs. GHQ28questionnaire results, (*n* = 65).

	Oral Sex	*n*	Mean	Median	SD	Min	Max	*p*-Value
**GHQ28 somatic symptoms**	No	21	7.57	7.00	3.572	3	18	0.838
	Yes	44	7.68	7.50	3.395	2	16	
**GHQ28 anxiety/insomnia**	No	21	7.38	8.00	3.827	2	17	0.714
	Yes	44	7.41	7.00	4.510	1	19	
**GHQ28 social functioning disorders**	No	21	9.62	8.00	4.873	6	28	0.712
	Yes	44	8.59	8.00	3.955	1	19	
**GHQ28 depression**	No	21	1.81	1.00	2.442	0	8	0.411
	Yes	44	1.27	0.00	2.171	0	9	
**Mell–Krat total**	No	20	49.35	49.50	8.152	31	63	0.080
	Yes	43	53.30	55.00	8.967	29	72	
**SSS-W contentment**	No	21	20.90	23.00	4.369	11	27	0.933
	Yes	44	20.66	22.00	4.951	10	27	
**SSS-W communication**	No	21	20.81	22.00	5.326	10	30	0.043
	Yes	44	23.70	24.00	4.688	12	30	
**SSS-W compatibility**	No	21	23.95	25.00	4.883	12	30	0.709
	Yes	44	24.34	25.00	4.918	12	30	
**SSS-W total**	No	21	65.67	68.00	12.228	40	86	0.261
	Yes	44	68.70	72.50	12.115	44	86	

## Data Availability

Data are contained within the article.
